# Clinical Efficacy and Microbiome Changes Following Fecal Microbiota Transplantation in Children With Recurrent Clostridium Difficile Infection

**DOI:** 10.3389/fmicb.2018.02622

**Published:** 2018-11-02

**Authors:** Xiaolu Li, Xuefeng Gao, Hui Hu, Yongmei Xiao, Dan Li, Guangjun Yu, Dongbao Yu, Ting Zhang, Yizhong Wang

**Affiliations:** ^1^Department of Gastroenterology, Hepatology and Nutrition, Shanghai Children’s Hospital, Shanghai Jiao Tong University, Shanghai, China; ^2^Shenzhen University General Hospital, Shenzhen, China; ^3^Shenzhen University Clinical Medical Academy, Shenzhen, China; ^4^Shenzhen Hoiracle Bio-Tech Co., Ltd., Shenzhen, China

**Keywords:** fecal microbiota transplantation, recurrent clostridium difficile infection, gut microbiota, 16S rRNA gene sequencing, children

## Abstract

Fecal microbiota transplantation (FMT) has been shown as an effective treatment for recurrent clostridium difficile infection (RCDI) in adults. In this study, we aim to evaluate the clinical efficacy of FMT in treating children with RCDI, and explore fecal microbiota changes during FMT treatment. A total of 11 RCDI subjects with a median age of 3.5 years were enrolled in this single-center prospective pilot study. All patients were cured (11/11, 100%) by FMT either through upper gastrointestinal tract route with a nasointestinal tube (13/16, 81.2%) or lower gastrointestinal tract route with a rectal tube (3/16, 18.8%). The cure rate of single FMT was 63.6% (7/11), and 4 (4/11, 36.4%) cases were performed with 2 or 3 times of FMT. Mild adverse events were reported in 4 children (4/11, 36.4%), including transient diarrhea, mild abdominal pain, transient fever and vomit. Gut microbiota composition analysis of 59 fecal samples collected from 34 participants (9 RCDI children, 9 donors and 16 health controls) showed that the *alpha* diversity was lower in pediatric RCDI patients before FMT than the healthy controls and donors, and fecal microbial community of pre-FMT samples (*beta* diversity) was apart from that of healthy controls and donors. No significant differences in *alpha* diversity, *beta* diversity or phylogenetic distance were detected between donors and healthy controls. Both the richness and diversity of gut microbiota were improved in the pediatric RCDI patients after FMT, and the bacteria community was shifted closer to the donor and healthy control group. Furthermore, FMT re-directed gut microbiome functions of pediatric RCDI toward a health state. Our results indicate that it is safe and tolerant to use FMT in treating pediatric RCDI. FMT shifted the gut microbiome composition and function in children with RCDI toward a healthy state.

## Introduction

*Clostridium difficile* (CD) is a leading causative pathogen of antibiotic-associated and healthcare-associated infective diarrhea. CD infection (CDI) is determined by the presence of symptoms (diarrhea) and either a detection of toxin producing *C. difficile* in stool, or findings of pseudomembranous colitis by colonoscopy or histopathology (McDonald et al., 2018). Due to the emergence of more virulent CD strains, the prevalence and severity of CDI in children increased in past decades (Khanna et al., 2013; Sammons and Toltzis, 2013; Wendt et al., 2014; Gupta et al., 2016; Spigaglia et al., 2017). Clinical treatment with metronidazole, vancomycin or fidaxomicin is successful in most of CDI subjects, however, recurrence after completion of a first treatment course ranges from 15 to 20%, and increases up to 45–60% after the first recurrence (McFarland et al., 2002; Kelly and LaMont, 2008; Brandt, 2013). Studies revealed that the recurrence rate of pediatric RCDI after first-line treatment varied from 2.6 to 30% in different examined population, with the frequency of recurrence rising further after subsequent infections (Barbut et al., 2000; McFarland et al., 2002; Pai et al., 2012; Khanna et al., 2013; Nicholson et al., 2015). Conventional antibiotic treatment is less ineffective for refractory, recurrent and complicated CDI, and new therapeutic approaches are needed for managing RCDI.

The normal intestinal microbiome plays an important physiological role in the prevention of infection, maintenance barriers, immunity, metabolism, and nutrition of the host (Lloyd-Price et al., 2016). It has been shown that either colonization or infection with CD is associated with reduced microbiota diversity in human gut (Samarkos et al., 2018). Fecal microbiota transplantation (FMT), first described in 1958 (Eiseman et al., 1958), is known as the administration of a solution of fecal matter from a donor into the intestinal tract of a recipient in order to cure a specific pathology (Aroniadis and Brandt, 2013; Mattner et al., 2016), which has been received increasing attention as an effective treatment for RCDI with cure rates of 90–100% in adults (Drekonja et al., 2015). However, the data of FMT for pediatric RCDI are rare. This study aimed to evaluate the clinical efficacy and safety of FMT and examine the influence of FMT on the diversity, structure, and function of gut microbiome in pediatric RCDI patients.

## Materials and Methods

### Study Cohort

Eleven children with RCDI underwent FMT from Shanghai Children’s Hospital, China, between September 2014 and December 2017, were enrolled to the study cohort. This study was approved by the Regional Ethical Review Board in Shanghai Children’s Hospital. Data including demographics, clinical history, CDI treatment history, and clinical response to FMT were collected and analyzed. Clinical cure was defined as the resolution of diarrhea attributable to CDI, and without recurrence of CDI in 3 months after FMT. Nine stool donors and 16 health individuals were included in the study for gut microbiome analysis. Written informed consent was obtained from parents of all pediatric subjects, donors, and health controls.

### FMT Procedure

Stool donors including healthy family members and unrelated volunteers were screened based on medical history and laboratory testing. Donor being on prescription drugs, antibiotic exposure in recent 3 months, body mass index >30 were excluded. Donors have history of malignancy, chemotherapy, chronic systemic or gastrointestinal disease, or functional disorders were also excluded. Informed consents were obtained before screening procedures. Laboratory testing including blood tests of syphilis, HIV, hepatitis A, B, and C, and stool tests including CD toxin, bacterial culture for enteric pathogens, ova and parasites, Giardia antigen and cryptosporidium antigen were performed. Fresh stool from donor was collected and blended using 200–250 mL sterilized saline per 150 g stool at high speed for 2–3 min. Stool suspension was filtered by 2 layers of medical gauze to remove large particles. Stool filtrate was drawn into 50 mL syringes for immediate FMT, or collected in 50 mL tubes frozen in -80°C for further FMT. Recipients were stopped antibiotic prescription for CDI 48 h prior to the FMT procedure. FMT was initially performed using an upper route to the distal duodenum with a nasal jejunal tube, and 50–100 mL of a processed donor stool suspension was instilled during the procedure. In the case of a failure of the upper route for FMT, a second FMT was performed via a lower route by retention enema with 100–200 mL donor stool suspension. If clinical symptoms were not improved after 2 days of FMT, the second FMT was administrated. Clinical efficacy and adverse events were assessed at 1, 2 weeks, and 3 months after FMT.

### Fecal Microbiome Analysis

Stool samples of patients were collected 1 day before FMT, and on days 1, 3, 7, 30+ after FMT. All samples were stored at -80°C until shipping to the HRK-biotech lab for DNA extraction and sequencing. The isolation methods are described as previously (Wang et al., 2018). Isolated genomic DNA was amplified for the V3–V4 hypervariable regions of the 16S rRNA gene and sequenced using an Illumina Miseq platform. USEARCH (Edgar, 2010) *cluster_otus* command was employed to filter chimeric sequences and generate cluster operational taxonomic units (OTUs) based on 97% nucleotide similarity. Taxonomic assignment was performed by using the Ribosomal Database Project (RDP) classifier. The statistical analysis was performed with SHAMAN (shaman.c3bi.pasteur.fr). OTU counts were normalized by taking into account the confounding effect of sex and age. Overall differences in microbiome structure were evaluated through Principal Coordinate Analysis (PCoA) to Bray-Curtis distance. A generalized linear model (GLM) with contrast vectors was defined to evaluate the significant differences in the gut microbiome compositions among the sample groups. The resulting *p*-values were adjusted for multiple testing according to the Benjamini-Hochberg procedure. PICRUSt (Phylogenetic Investigation of Communities by Reconstruction of Unobserved States) was applied to predict the metagenomes from 16S rRNA data (Langille et al., 2013). The organism-level microbiome phenotypes were predicted by using BugBase (Ward et al., 2017).

## Results

### Characteristics of Pediatric RCDI

The characteristics of the 11 patients were summarized in Table 1. Of all these patients (5 boys and 6 girls), the median age at the time of FMT was 3.5 years (range, 0.5–12 years). All subjects were treated with 1–2 episodes of oral antibiotics before FMT, 7 patients (7/11, 63.6%) were given oral vancomycin, and all 11 (11/11, 100%) subjects received metronidazole treatment. Seven children (7/11, 63.6%) were diagnosed as pseudomembranous colitis based on the results of endoscopy. The main manifestations of abdominal CT scan were effusion and pneumatosis in 10 (10/11, 90.9%) patients.

**Table 1:** Characteristics of RCDI children treated with FMT.

	n (%)
Gender (Female)	6 (54.5)
Median age at FMT (years, range)	3.5 (0.5–12)
**Clinical symptoms**	
Diarrhea	7 (63.6)
Bloating	1 (9.1)
Abdominal pain	1 (9.1)
Blood stool	2 (18.2)
Median duration of symptoms in months (range)	4 (1–12)
**Endoscopy**	
Pseudomembranous colitis	7 (63.6)
Inflammation	4 (36.4)
**Abdominal CT scan**	
Effusion	10 (90.9)
Pneumatosis	10 (90.9)
**Antibiotics received for CDI beforeFMT**	
Metronidazole	11 (100)
Vancomycin	7 (63.6)


*RCDI, recurrent clostridium difficile infection; FMT, fecal microbiota transplantation.*

### Clinical Responses to FMT

Total 16 FMT were performed, 81.2% (13/16) using an upper route with a nasointestinal tube and 18.8% (3/16) through lower route by retention enema. Six donors (6/11, 54.5%) are parents of children and 5 (5/11, 45.5%) are unrelated donors. Seven (7/11, 63.6%) children were cured by single FMT, and 4 (4/11, 36.4%) children were cured with 2–3 times of FMT (Table 2). There was no recurrence of diarrhea during 3 months after FMT, and CD toxin tests were negative. There is no difference in clinical response to FMT related to the sources and ages of donors.

**Table 2:** Characteristic and clinical response of FMT for RCDI children.

	n (%)
**Donors’ relationship to patient**	
Parent	6 (54.5)
Unrelated volunteer	5 (45.5)
Donors’ gender (Female)	4 (36.4)
Median age of donors (years, range)	31 (27–49)
Number of previous CDI episodes	1.5 (1–2)
**Route of FMT, n (%)**	
Nasal jejunal tube	13 (81.2)
Retention enema	3 (18.8)
Cure of CDI, n (%)	11 (100)
Single	7 (63.6)
Multiple (2–3 times)	4 (36.4)
Time from FMT to resolution of diarrhea (days)	1 (1–2)
Adverse events, n (%)	4 (36.4)
Transient diarrhea	1 (9.1)
Fever	1 (9.1)
Transient mild abdominal	1 (9.1)
Vomit	1 (9.1)
Median follow-up time, in months (range)	18 (9–36)


*RCDI, recurrent clostridium difficile infection; FMT, fecal microbiota transplantation.*

There were no serious adverse events reported after 3 months of FMT. Four children (4/11, 36.4%) were reported with mild and self-limited adverse events. Transient diarrhea was reported in 1 patient on the day of FMT, and disappeared after 2 days. Transient mild abdominal pain was reported in 1 child immediately after the procedure. One patient had transient fever and returned to normal on the time of second FMT. In addition, 1 child had vomit during FMT procedure. All subjects were followed up in every 3 months after FMT by phone call. The average follow-up time was 18 (range: 9–36) months (Table 2). No recurrence of CDI and FMT-related adverse events were reported by the parents of subjects.

### FMT Corrected Dysbiosis and Re-established Intestinal Homeostasis in Pediatric RCDI

**FIGURE 1:**
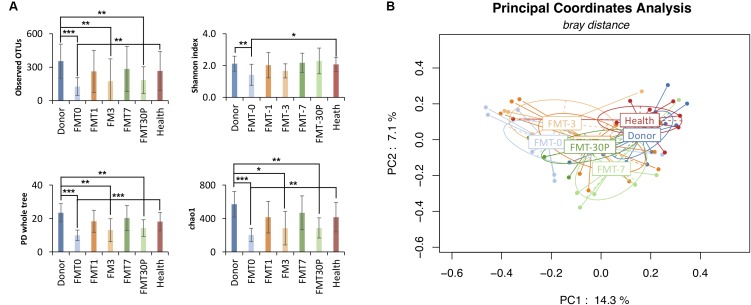
FMT increases bacterial diversity of pediatric RCDI, and re-directs the gut microbiome toward a health state. **(A)** *Alpha* diversity was calculated using the number of overserved OTUs, Shannon index, PD whole tree, and chao1, with ^∗^*p* < 0.05, ^∗∗^*p* < 0.01, and ^∗∗∗^*p* < 0.001 (Student *T*-test). **(B)** Principal coordinate analysis (PCoA) profile of microbial diversity across all samples using Bray-Curtis distance. Each dot stands for one sample, and the ellipses stand for sample groups. The clustering based on microbial distribution is significant (analysis of variance using PERMANOVA test, *p*-value = 0.001).

For characterizing gut microbiota associated with pediatric RCDI, a total of 59 fecal samples were collected from 34 participants including 9 RCDI children, 9 donors and 16 health individuals. Thirty-eight fecal samples from RCDI children were included: 9 at baseline (prior to FMT, FMT0) and 29 at various days (1, 3, 7, and 30+ days) after FMT. For children treated with multiple FMT, pre-FMT fecal samples were collected before the first time of FMT, and post-FMT samples were collected from post days of last FMT procedure. Each donor (*n* = 9, between the ages of 27 and 49 years with a mean of 31 years) and health subjects (*n* = 16, between the ages of 4 and 17 years with a mean of 12 years) provided a single stool sample. The *alpha* diversity was found lower in pediatric RCDI patients before FMT treatment (herein after termed FMT0) than the healthy controls and donors (Figure 1A). Analysis of the *beta* diversity calculated on the Bray-Curtis dissimilarity, unweighted and weighted UniFrac distances (Figure 1B) revealed that fecal microbial community of pre-FMT samples apart from that of healthy controls and donors. No significant differences in *alpha* diversity, *beta* diversity or phylogenetic distance were detected between donors and healthy controls.

**FIGURE 2:**
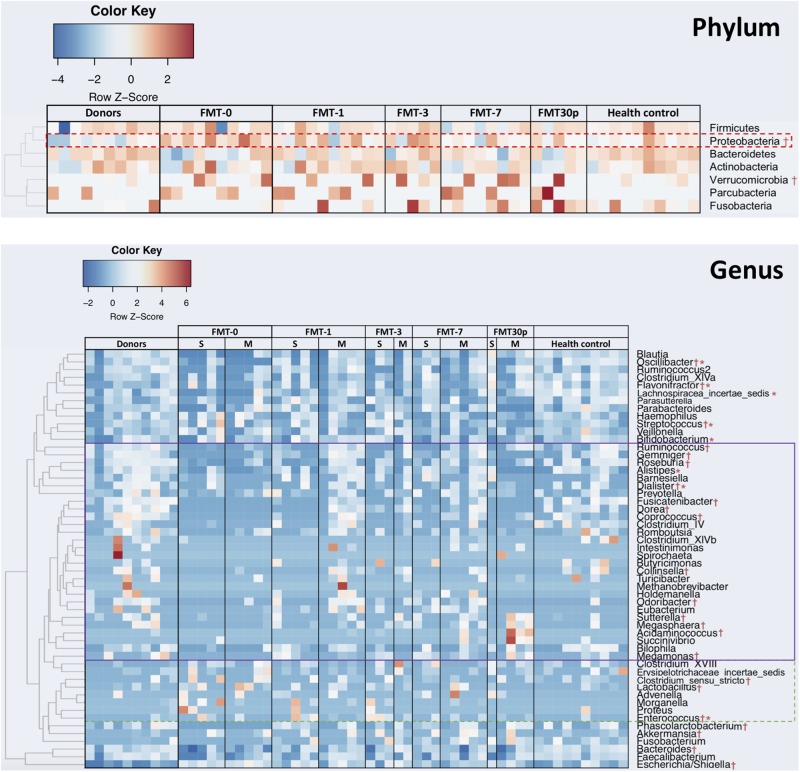
FMT shifts the gut bacterial composition in children with RCDI toward a healthy state. Phyla and genera that were statistically different in abundance between the FMT-0 and donor/health control groups are identified by † (*p* < 0.05), and between the single-FMT-0 and multiple-FMT-0 are identified by ^∗^ (*p* < 0.05). Heatmap is color-coded based on row z-scores. Genera having increased and decreased relative abundances after FMT are highlighted by a solid and dashed outlines, respectively. S, Single-FMT; M, Multiple-FMT.

Inter-group comparisons of taxonomic profiles revealed that gut microbiome from patients prior to FMT therapy exhibited alterations in the abundance of several taxa. At the phylum level, Proteobacteria was enriched in the children with RCDI (Figure 2). At the genus level, relative abundances of *Escherichia/Shigella*, *Lactobacillus*, *Streptococcus*, *Enterococcus*, *Akkermansia*, *Clostridium*_*sensu*_*stricto*, and *Flavonifractor* were significantly higher in the pre-FMT samples, while *Phascolarctobacterium*, *Fusicatenibacter*, *Ruminococcus*, *Coprococcus*, *Dorea*, *Gemmiger*, *Collinsella*, *Megamonas*, *Roseburia*, *Megasphaera*, *Bacteroides*, and *Oscillibacter* were significantly reduced compared to the controls and/or donors.

Following FMT treatment (herein after termed FMT-x, which indicated the samples were collected at x days after FMT, FMT-30P group includes samples collected at 30 and plus days after FMT), the bacterial diversity of intestinal microbiota was increased in the pediatric RCDI patients, and was maintained through the study (Figure 1A). PCoA showed that the bacterial communities of the FMT-1, -7, and -30P samples were shifted toward the healthy control and donor groups (Figure 1B). Statistical analysis of bacterial taxa revealed that the fecal microbiota of the pediatric RCDI patients was shifted toward a healthy composition by adopting a donor-like configuration after FMT (Figure 2). In specific, the relative abundances of Proteobacteria phylum declined significantly 7 days after FMT (Figure 2). At the genus level, some bacteria (indicated by solid outline in Figure 2) that depleted in the RCDI patients were uplifted after FMT. Some genera (indicated by dashed outline in Figure 2) that enriched in the pediatric RCDI patients were reduced following FMT and maintained during the study period. The microbiome composition changed rapidly after FMT, however, some bacteria drifted toward the baseline after 30 days post-FMT, such as *Escherichia/Shigella*, *Flavonifractor*, *Fusicatenibacter*, *Dorea*, *Gemmiger*, and *Collinsella*.

In our study, all 11 children with RCDI were cured, albeit a proportion (7/11) achieved remission after single FMT while remission occurred after multiple FMT for the others (4/11) (Table 2). We thus the patients into single-FMT and multiple-FMT and compared their gut bacterial composition. The *alpha* diversity was significantly lower in the pediatric patients achieved cure after multiple FMT compared to the donors and healthy controls (Supplementary Figure S1). Taxonomically, the Actinobacteria phylum and *Lachnospiracea incertae sedis* genus were significantly more abundant in the patients that achieved cure with multiple FMT while the genera *Bifidobacterium*, *Enterococcus*, *Alistipes*, *Dialister*, *Streptococcus*, *Oscillibacter*, and *Flavonifractor* were enriched in patients with symptom improvement sooner after a single FMT (Figure 2).

### FMT Re-directed Gut Microbiome Functions of Pediatric RCDI Toward a Health State

**FIGURE 3:**
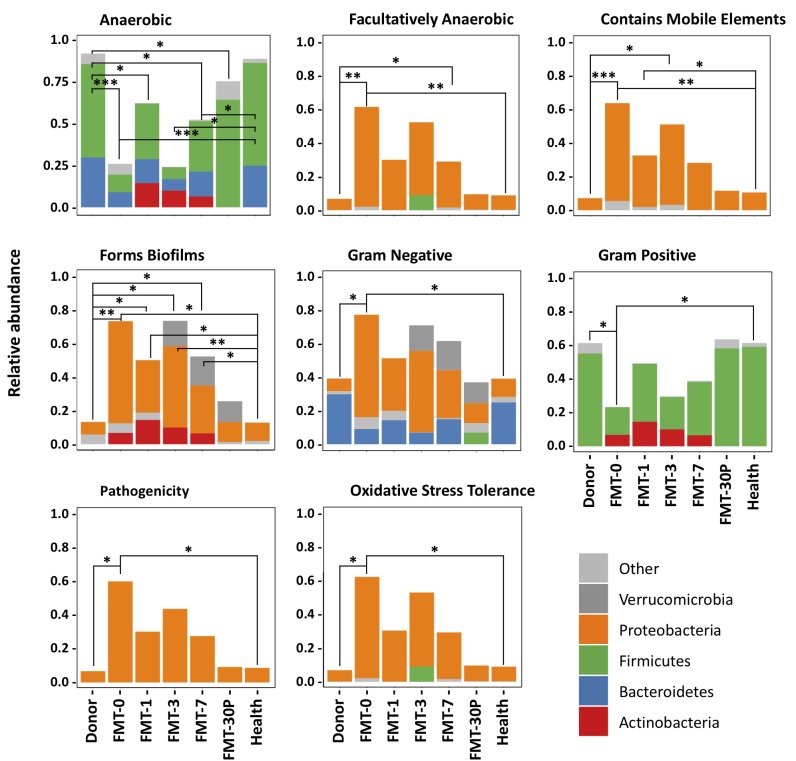
BugBase predicts microbial community phenotypes and the corresponding contributions from different phyla. Statistical significance was determined by Pairwise Mann-Whitney-Wilcoxon tests. FDR-corrected pairwise *p*-value ranges are: ^∗^*p* < 0.05, ^∗∗^*p* < 0.01, and ^∗∗∗^*p* < 0.001.

We used BugBase to analyze community-wide phenotypes of the stool microbiome. We observed that the microbiome phenotypes were similar between the donors and health controls, indicating a common healthy state was shared by these individuals (Figure 3). The gram-positive species and gene functions associated with anaerobiosis were the lowest in the pre-FMT samples, and mainly due to some reduced taxa that belong to Firmicutes. The gram-negative taxa and gene functions associated with facultative anaerobiosis, biofilms forming, mobile element content, pathogenesis, and oxidative stress tolerance were enriched in the pediatric RCDI subjects prior to FMT, and the major contributors came from Proteobacteria. After FMT, most of the functional capacities were re-directed rapidly to a healthy level and maintained through the study. However, the proportions of anaerobic bacteria and that associated with biofilm forming changed slowly after FMT, and approached a healthy level 30 days (or longer) after treatment.

## Discussion

As an emerging and promising treatment in RCDI, FMT was proved to be more effective than conventional antibiotics treatment (van Nood et al., 2013; Drekonja et al., 2015). FMT has been strong recommended for adult patients with multiple recurrences of CDI who have failed appropriate antibiotic treatments for its high success rate (McDonald et al., 2018). However, study focused on FMT for pediatric RCDI was still limited. A recent review summarized the published data in FMT for pediatric RCDI showed that 89% (40/45) of patients had symptom improvement (Chen et al., 2017). An emerging study showed that 15 children with RCDI were cured by FMT, including five children with underlying IBD (Fareed et al., 2018). In the present study, a total of 11 RCDI subjects with a median age of 3.5 years were cured by FMT. The cure rate of single FMT was 63.6% (7/11), and 36.4% (4/11) cases performed with 2 or 3 times of FMT. The lower cure rate of single FMT may due to the low feces dose infused to the gut or short retention time. There was no recurrence of diarrhea during the 6–36 months of follow up after FMT. No serious adverse event was observed after 3 months of FMT. Four children (36.4%) were reported with mild and self-limited adverse events, including transient diarrhea, transient mild abdominal pain, transient fever and vomit during FMT procedure. Our data showed that the cure rate of pediatric RCDI by FMT was comparable to adults (Drekonja et al., 2015), however, due to the limited number of pediatric RCDI treated with FMT, it is important to study the effect of FMT on pediatric RCDI in large cohort and well designed randomized controlled trial in the future.

Fecal microbiome profiles of pediatric RCDI patients were analyzed and compared with the donors and a control group of healthy children. Our data showed that the fecal microbial community of pre-FMT samples apart from that of healthy controls and donors. *Alpha* diversity in pediatric RCDI patients prior to FMT, especially those who occurred remission after multiple FMT, was much lower compared with both the healthy controls and the donors. Inter-group comparisons of taxonomic profiles revealed that samples from patients prior to FMT exhibited alterations in the gut microbiome composition. Interestingly, we also observed that seven genera were enriched in the patients that were cured by a single FMT while the *Lachnospiracea incertae sedis* genus was more abundant in those achieved cure after multiple FMT. Previous study has shown that the genus *Lachnospiracea incertae sedis* is significantly enriched in CD-associated disease and irritable bowel syndrome (IBS) patients (Frank et al., 2007). In fact, members of the family Lachnospiraceae are able to induce an elevated immune reactivity in Crohn’s disease patients and a subset of IBS patients through expressing highly antigenic flagellins (Duck et al., 2007; Schoepfer et al., 2008). Nevertheless, data characterizing gut microbiota changes after FMT in children are limited. In our study, the microbiota diversity increased in fecal samples of RCDI children after FMT, which agrees with previous findings (Walia et al., 2014; Hourigan et al., 2015). Further analysis of bacterial taxa revealed that the fecal microbiota of the pediatric RCDI patients after FMT was shifted toward a healthy composition by adopting a donor/health-control-like structure. In addition, the gut microbiome functions were re-directed by FMT to a healthy state. Although the abundances of some taxa moved toward baseline at 30 and more days post-FMT, the gut microbiome functions maintained as a similar status as the donors and healthy controls.

## Conclusion

In this study, all 11 pediatric RCDI subjects were cured by FMT. FMT increased the diversity of the gut microbiota in children with RCDI, and shifted their microbiota composition and functions toward that of the donors and health controls. FMT is likely safe and tolerant in treating children with RCDI.

## Ethics statement

This study was carried out in accordance with the recommendations of the Declaration of Helsinki, World Medical Association. The study protocol was approved by the Regional Ethical Review Board of Shanghai Children’s Hospital. Written informed consent was obtained from parents or legal guardians of all children eligible for study enrollment.

## Author Contributions

YW and TZ designed the study. XL, XG, and DY interpreted the data, created figures, and wrote the manuscript. HH, XL, YX, DL, and GY analyzed and interpreted the patient data regarding the RCDI. All authors read and approved the final manuscript.

## Conflict of Interest Statement

The authors declare that the research was conducted in the absence of any commercial or financial relationships that could be construed as a potential conflict of interest.

## Supplementary Material

Figure S1 Biodiversity of gut bacterial community is lower in pediatric RCDI patients that achieved cure after multiple FMT. *Alpha* diversity was calculated using the number of overserved OTUs, Shannon index, PD whole tree, and chao1. *P*-value ranges are: ^∗^*p* < 0.05, ^∗∗^*p* < 0.01, ^∗∗∗^*p* < 0.001 (Student *T*-test).
