# Supplementary figures and images for: Clinical Efficacy and Microbiome Changes Following Fecal Microbiota Transplantation in Children With Recurrent Clostridium Difficile Infection
Source: Front Microbiol. 2018 Nov 2;9:2622. doi: 10.3389/fmicb.2018.02622 (PMC6224514; doi:10.3389/fmicb.2018.02622)

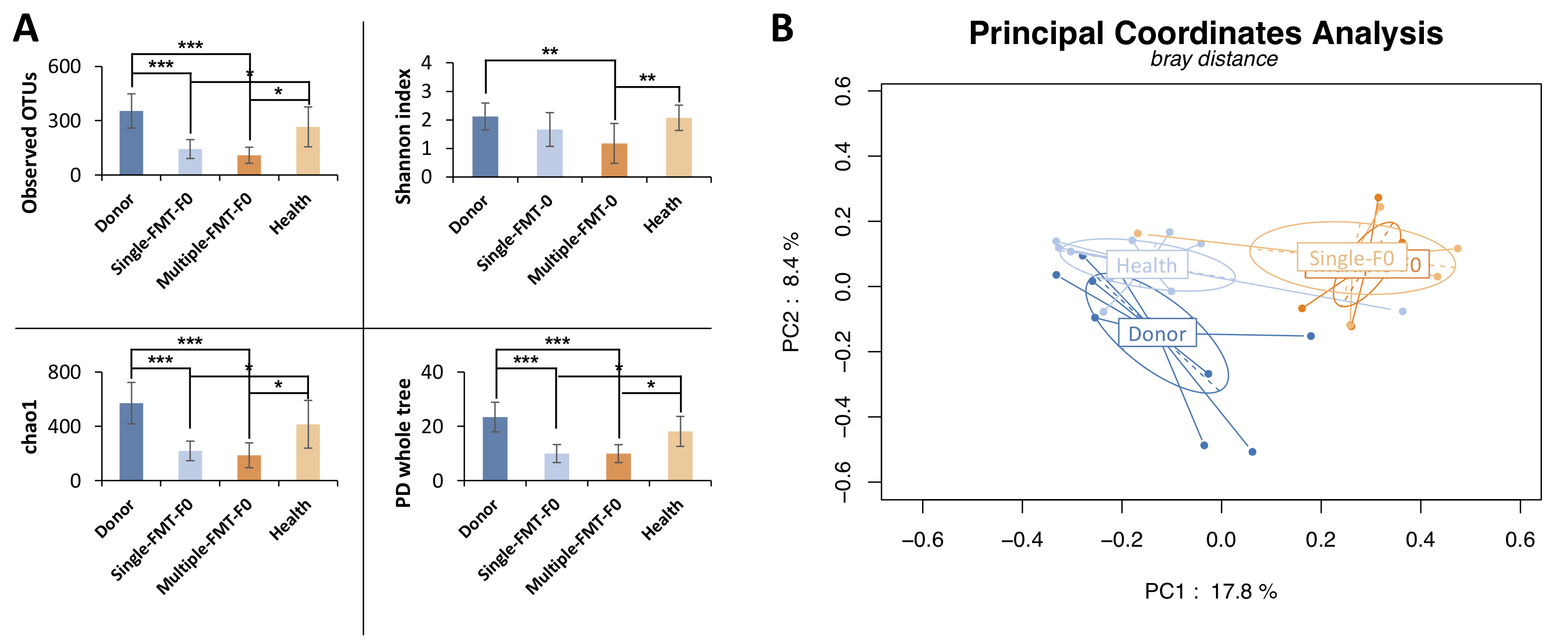

Supplement: Figure S1 — Biodiversity of gut bacterial community is lower in pediatric RCDI patients that achieved cure after multiple FMT. Alpha diversity was calculated using the number of overserved OTUs, Shannon index, PD whole tree, and chao1. P-value ranges are: ∗p < 0.05, ∗∗p < 0.01, ∗∗∗p < 0.001 (Student T-test). [file Image_1.TIFF]
